# Erxian decoction, a famous Chinese medicine formula, antagonizes corticosterone-induced injury in PC12 cells, and improves depression-like behaviours in mice

**DOI:** 10.1080/13880209.2020.1765812

**Published:** 2020-06-01

**Authors:** Lan Zhang, Yue Yang, Lei Di, Jun-long Li, Ning Li

**Affiliations:** School of Pharmacy, Anhui Medical University, Hefei, PR China

**Keywords:** Neurotransmitter, cell apoptosis, behavioural test

## Abstract

**Context:**

In folk medicine, erxian decoction (EXD) is used to treat perimenopausal syndrome in women. It is also used clinically to treat depression, but the mechanism remains unknown.

**Objectives:**

To investigate the neuroprotective effect of EXD, and its antidepressant potential.

**Materials and methods:**

ICR mice were treated with EXD (0.5, 1.5 and 4.5 g/kg i.g.) and fluoxetine (6.0 mg/kg i.g.) for 10 days. On day 10 of the treatment, depression-like behaviour was induced by reserpine (2.5 mg/kg injected i.p.), and after 24 h of reserpine administration, it was assessed using the tail suspension and forced swimming tests. MTT assay, lactate dehydrogenase test, flow cytometry analysis, Hoechst staining and western blotting were used to assess the apoptosis of PC12 cells. Apoptosis proteins and neurotransmitter were tested *in vitro* and *in vivo*, respectively.

**Results:**

MTT assay results showed corticosterone prevented cell growth, but EXD at concentrations of 100, 200 and 400 μg/mL restored cell viability (EC_50_: 204.016 μg/mL). EXD decreased lactate dehydrogenase leakage from 63.48 to 43.60 U/L, and upregulated expression of Bcl-2 while the expression of Bax, caspase-3 and caspase-8 were decreased *in vivo* and *in vitro*. Moreover, EXD improved depression-like behaviour in mice, and 4.5 g/kg EXD treatment increased the secretion of serotonin, dopamine and norepinephrine by 67.44, 28.12 and 42.12 pg/mg, respectively, in hypothalamus compared to that of reserpine group.

**Discussion and conclusions:**

EXD demonstrated neuroprotective effects and improved depression-like behaviour in mice. Further research should be focussed on the mechanism of the active components in EXD.

## Introduction

Depression is a common mental disorder characterized by lasting low moods, lack of motivation, feelings of despair and anhedonia (Baquero and Martin 2015). According to Malhi and Mann (2018), depression will be the primary leading disease in the world by 2030, and the major depressive disorder (MDD) is among the top three causes of years lived with disability, which impose a heavy burden on the individual, family and society (Vos et al. 2016). Although several classes of antidepressants have been developed for clinical application, the existing drugs are far from satisfactory due to the many side effects (Zanos et al. 2016). Besides, only approximately 30% of MDD patients respond to current antidepressants, and about 70% of those who respond do not achieve full remission (Thase 2013). Thus, efficacious agents, which have no side effects, are urgently needed.

Owing to the side effects of synthetic antidepressants, herbal medicines, such as *Hypericum perforatum* L. (Clusiaceae), have been accepted as a safer option for the treatment of depression (Sarris 2018). In the theory of traditional Chinese medicine (TCM), the kidney and brain are closely related to each other in function, and kidney-tonifying medicines will benefit brain health. Some TCM formulas have demonstrated therapeutic effects on depression (Xu et al. 2004). One such formula is erxian decoction (EXD).

Erxian decoction is a well-known Chinese medicine formula that was developed by Zhang Bo-Na in the early 1950s (Li et al. 2007). The formula consists of six Chinese medicinal herbs, namely rhizome *Curculigo orchioides* Gaertn. (Hypoxidaceae), Herba *Epimedium brevicornu* Maxim. (Berberidaceae), radix *Morinda officinalis* F. C. How (Rubiaceae), radix *Angelica sinensis* (Oliv.) Diels (Apiaceae), cortex *Phellodendron chinense* C. K. Schneid. (Rutaceae) and rhizome *Anemarrhena asphodeloides* Bunge (Anthericaceae) (Wang et al. 2015). EXD was created to remedy the syndromes of *Shen-yang* and *Shen-yin* deficiency (where ‘*shen*’ means kidney in Chinese) and harmonise the ‘*yin-yang*’ balance. In the EXD formula, *C. orchioides*, *E. brevicornu* and *M. officinalis* invigorate *Shen-yang*, while *A. asphodeloides* benefits *Shen-yin* (Li et al. 2007). EXD was originally designed for the treatment of menopausal hypertension in female patients (Zhang 1958). However, after 60 years of clinical practice and experimental research, the scope of EXD in clinical application was extended to many chronic diseases (Li et al. 2007).

Today, EXD is an empirical TCM prescribed for female perimenopausal syndromes (PMS), including perimenopausal depression. In folk medicine, EXD has been used clinically to treat depression and yielded satisfactory therapeutic effects (Guo 1988). A network pharmacological analysis has also indicated that EXD possesses the potential to treat neurological diseases (Li et al. 2007). However, until now there has been no detailed study on the neuroprotective and antidepressant effects of EXD.

Reserpine (Res), an alkaloid extracted from the root of *Rauwolfia serpentine* which was first introduced to modern medicine in the mid-1940s, is one of the earliest drugs used to treat hypertension (Mashour et al. 1998). A recent study suggests that reserpine depletes the neuronal storage granules of biogenic amines in the brains of rodents, and causes a clinically significant depression-like state (Minor and Hanff 2015). Therefore, reserpine was designed to induce depressive symptoms in mice in our experiment. Fluoxetine (Flu), the most widely used antidepressant, increases serotonergic neurotransmission through selective inhibition of neuronal reuptake of serotonin (Amitai et al. 2016). Fluoxetine also plays an important role in neurogenesis and neuroplasticity, which may have significant effects on neurotrophic disease and increase neuronal energy supply (Levy et al. 2019). Thus, we chose fluoxetine as a positive drug in this experiment. This study evaluates the neuroprotective effects of EXD on corticosterone (Cort)-injured PC12 cells *in vitro* and confirms the antidepressant-like effects in despaired mice and reserpine-induced mouse models *in vivo*.

## Materials and methods

### Materials and drugs

The following raw medicinal herbs for EXD were purchased from Anhui Zhiyuan Traditional Chinese Medicine Co. Ltd. (Bozhou, China): *C*. *orchioides* (place of origin: Sichuan, China; batch number: 180301), *E*. *brevicornu* (place of origin: Shanxi, China; batch number: 180302), *M*. *officinalis* (place of origin: Guangdong, China; batch number: 180401), *A*. *sinensis* (place of origin: Gansu, China; batch number: 180402), *P*. *orientalis* (place of origin: Sichaun, China; batch number: 180407), *A*. *asphodeloides* (place of origin: Hebei, China; batch number: 180306). Antibodies against Bax and Bcl-2, cleaved caspase-3 and caspase-8 were procured from Abcam (Cambridge, MA), Cell Signalling Technology (Danvers, MA) and Elabscience Biotechnology Company (Wuhan, China), respectively. Dulbecco’s modified Eagle’s medium (DMEM) was procured from Thermo Fisher HyClone (Logan, UT). Foetal bovine serum was purchased from Evergreen Biological Engineering Materials Corporation (Hangzhou, China). Horse serum was purchased from Biological Industries Corporation (Beijing, China). MTT was purchased from Beijing Solarbio Science and Technology Corporation (Beijing, China). Hoechst 33258 staining kit was procured from Beyotime Institute of Biotechnology (Shanghai, China). Fluoxetine, reserpine and corticosterone were purchased from Aladdin Industrial Corporation (Boston, MA). Enzyme-linked immunosorbent assay (ELISA) kits were purchased from Jiangsu Meimian Industrial Corporation (Jiangsu, China). The standards for mangiferin (98%), ferulic acid (99%), berberine (99%), epimedin C (98%) and rubiadin (98%) were purchased from the Chinese National Institute for Control of Pharmaceutical and Biological Products (Beijing, China). Orcinol (98%), 2, 6-dimethoxybenzoic acid (98%) and curculigoside (98%) were prepared by our group. Other chemicals were purchased from Sigma-Aldrich (St. Louis, MO) unless otherwise indicated.

### Preparation of EXD

The herbal materials were authenticated by Prof. Kai-Jin Wang from the School of Life Science, Anhui University, China. Voucher specimens (No. 2018001-6) were deposited at the School of Pharmacy, Anhui Medical University, China. The EXD extract was prepared according to previously reported methods (Wang et al. 2017) with slight modifications. Briefly, the raw herbs of *C*. *orchioides*, *E*. *brevicornu*, *M*. *officinalis*, *A*. *sinensis*, *P*. *orientalis* and *A*. *asphodeloides* were mixed at the ratio of 12:12:10:10:9:9. The mixture (500 g) was ground into a powdered form, and the constituents extracted with double-distilled water (5 L × 2) at 100 °C for 3 h. The mixed extract was filtered and concentrated at 50 °C, lyophilized to yield EXD powder, and then stored at −20 °C for subsequent use.

### High performance liquid chromatography (HPLC) analysis of EXD

The analysis of the constituents was performed on an Agilent HPLC (1260 infinity) equipped with a diode array detector (1260 variable wavelength detector) with an XBridge® C_18_ column (5 μm, 150 mm × 4.6 mm i.d., Shimadzu, JPN). The chromatographic separations were carried out on a mobile phase consisting of 0.1% acetic acid (A) and acetonitrile (B) using a gradient programmes of 5–5% (B) in 0–15 min, 5–20% (B) in 15–20 min, 20–40% (B) in 20–35 min and 40–50% (B) in 35–60 min at a flow rate of 1.0 mL/min and column temperature of 25 °C. The injection volume was 10 μL, and the UV detection wavelength was 351 nm.

The EXD extract (0.1 g) was placed in a volumetric flask with 10.0 mL of 95% methanol at 60 °C for 2 h, during which the mixture was subjected to ultra-sonication for 15 min twice. Authentic samples were prepared in methanol solution (1.0 mg/mL). All the samples were filtered through a 0.22 μm filter (Millipore) before they were injected into the HPLC. Peaks were confirmed using the UV absorption and retention times of the authentic samples.

### Cell culture

Rat pheochromocytoma cells, PC12 cells, were obtained from Nanjing Kaiji Biotech Company (Nanjing, Jiangsu, China). The cells were cultured in a medium containing 90% DMEM, 4.5% heat-inactivated horse serum, 4.5% foetal bovine serum and 1% penicillin–streptomycin. The cells were incubated at 37 °C in 95% humidity and 5% CO_2_. The culture medium was changed every 2 days. EXD was dissolved in DMSO and diluted with the 1640 medium before use.

### Cell viability assay

The neuroprotection of EXD against corticosterone-injured PC12 cells were assayed using the MTT method. Briefly, about 1 × 10^5^ PC12 cells/mL were placed in 96-well plates, and allowed to adhere for 24 h. The cells were then exposed to 200 μM corticosterone. EXD (100, 200 and 400 μg/mL) was added 1 h before corticosterone treatment. After 24 h of incubation, 20 μL of MTT (5 mg/mL) was added to each well, and the mixture was left to incubate for a further 24 h at 37 °C. During the final 4 h of incubation, 150 μL of DMSO was added to each well to dissolve the formazan crystals of viable cells. Readings were recorded at a wavelength of 570 nm using a Wallac 1420 ARVOsx microplate reader (Perkin-Elmer Life and Analytical Sciences, Inc., Boston, MA). Fluoxetine (10 μM) was used as a positive control. Cell viability is expressed as a percentage of the control.

### Cellular morphology observation

PC12 cells (5 × 10^4^) grown in 24-well plates were treated with different concentrations of EXD for 24 h with or without 200 μM corticosterone, and cellular morphological changes were observed using an inverted microscope (Nikon Eclipse TS100).

### Hoechst 33258 staining

Cells were seeded on a glass slide and pre-treated with different concentrations of EXD for 1 h with or without 200 μM corticosterone, and cultured for another 24 h. Next, they were washed twice with ice-cold PBS and fixed with 4% (v/v) formaldehyde in PBS for 20 min, washed with PBS again, stained with 1 mg/mL Hoechst 33258 in PBS at 37 °C for 15 min, and then viewed under a fluorescence microscope (Nikon) through a 420 nm filter at an excitation wavelength of 345 nm.

### Lactate dehydrogenase (LDH) leakage measurement

Cells pre-treated with EXD, and subsequently with or without corticosterone (200 μM), were assessed quantitatively for cytotoxicity by measuring the activity of LDH released from damaged cells into the culture medium. Briefly, cells were treated with 0.5% Triton X-100, and the media containing detached cells were collected and centrifuged. The supernatant was used for the assay of LDH activity. Enzyme activity was determined by using an assay kit according to the manufacturer’s instructions. LDH activity in cell culture medium (U/L) = (OD value in the assay group − OD value in the control group)/(OD value in the criteria group − OD value in the blank group) × standard concentration (0.2 mmol/L) × 5000.

### Flow cytometry used to detect apoptosis in PC12 cells

PC12 cells were seeded at a density of 1 × 10^5^ cells/well in six-well plates for 12 h, and then treated with different concentrations of EXD with or without corticosterone (200 μM) for 24 h, harvested with trypsin, washed twice with ice-cold PBS, re-suspended in FITC Annexin V and PI (5 μL each), and incubated for 15 min at room temperature in the dark before analysis with a FACSCalibur flow cytometer (BD Biosciences, CA) at an excitation wavelength of 480 nm.

### Assessment of PC12 proteins via Western blotting

PC12 cells (1.5 × 10^5^) grown in 24-well plates were treated with different concentrations of EXD for 24 h with or without 200 μM corticosterone. Next, they were washed twice with ice-cold PBS, dissolved in a lysis buffer containing 1% phenylmethylsulfonyl fluoride. Cell lysates were collected and centrifuged at 12,000 rpm for 12 min at 4 °C to remove the debris. Each protein sample was loaded and separated by 12% SDS-PAGE, and then transferred to a polyvinylidene difluoride membrane. Non-specific binding sites were blocked with 5% non-fat milk for 1 h. Samples were incubated with primary antibodies overnight at 4 °C, followed by another incubation with a secondary antibody for 1 h at room temperature. The protein bands were detected with an enhanced chemiluminescence system, and the grey value of the strips were analysed and counted by Image J programme, and the protein expression was normalised to β-actin.

### Animals

Male ICR mice (18–22 g) were obtained from the Experimental Animal Centre of Anhui Medical University. Animals were housed in groups of 6 per plastic cage (30 × 15 × 10 cm) with a 12 h light/dark cycle (lights on from 0700 to 1900 h), a constant temperature (21 ± 1 °C), and a relative humidity (55 ± 5%) for 1 week with free access to standard laboratory food and tap water to allow for adaption to the laboratory environment before the experiment. All procedures in this study were performed following the principles of Helsinki and were approved by the Ethics Committee and the Animal Experimental Committee of Anhui Medical University (Reference No. LLSC20190281).

### Animal grouping and drug administration

EXD and fluoxetine were suspended in 0.5% sodium carboxymethyl cellulose (CMC-Na) at specific concentrations before use. In the despair model, 40 mice were randomised into 5 groups according to their body weight (8 mice per group). The mice in the drug-treated groups were administered intragastrically (i.g.) with EXD (0.5, 1.5 and 4.5 g/kg) or fluoxetine (6.0 mg/kg) daily for 10 days. Control group mice received an equivalent volume of 0.5% CMC-Na. The final drug administration occurred 1 h before the forced swim test (FST) and tail suspension test (TST).

In the reserpine experiment, 48 mice were randomised into six groups according to their body weight (8 mice per group). The mice in the drug-treated groups were administered i.g. with EXD (0.5, 1.5 and 4.5 g/kg) or fluoxetine (6.0 mg/kg) daily for 10 days. Control group and reserpine group mice received an equivalent volume of 0.5% CMC-Na. All the animals, except those in the control group, were given intraperitoneal (i.p.) injections of reserpine (2.5 mg/kg, dissolved in 0.5% glacial acetic acid) 30 min after the final drug administration.

### Forced swim test

The test was carried out as described by Porsolt et al. (1977), with slight modifications. Briefly, the animals were subjected to 15 min of swimming for adaptation one day before the test. On the test day, each mouse was placed in a transparent glass vessel (25 cm in height, 14 cm in diameter) filled with 10 cm of water at 24 ± 2 °C. The total duration of immobility was measured during the last 4 min of a single 6 min test session. Mice were considered immobile when they did not attempt to escape except the movements necessary to keep their heads above the water.

### Tail suspension test

The TST was performed as described by Steru et al. (1985). Animals were placed in the laboratory for 2 h. Food and water was removed before the beginning of the experiment and throughout the process. Mice were suspended 5 cm above the floor using adhesive tape placed approximately 1 cm from the tip of the tail. The total duration of immobility was quantified during a test period of 6 min. Mice were considered immobile when they were completely motionless.

### Tests for reserpine-induced hypothermia, ptosis and akinesia

The tests for reserpine-induced ptosis, akinesia and hypothermia were performed as described by Bourin et al. (1983), with slight modifications. The mice were treated with reserpine (2.5 mg/kg, i.p.) 30 min after the administration of EXD or fluoxetine, the animals were placed on a shelf (20 cm above the tabletop), and the degree of ptosis of each animal was recorded and evaluated 1 h later according to the following rating scale: 0, eyes opened; 1, eyes one-quarter closed; 2, eyes half-closed; 3, eyes three-quarters closed; 4, eyes completely closed. To measure akinesia, mice were placed at the centre of a circle (7.5 cm, diameter) 1 h after the injection of reserpine and considered akinetic (i.e., present or not) if they remained within the circle after 15 s.

### Tissue

After anaesthesia with ether and sacrifice, the hippocampus and hypothalamus of each mouse were rapidly removed, divided and frozen in liquid nitrogen at −200 °C. The hippocampus was homogenized in a radioimmunoprecipitation assay buffer. Before homogenization, a protease inhibitor cocktail and the phosphatase inhibitor PhosSTOP were added. After homogenization, tissue lysates were collected and centrifuged at 12,000 rpm for 12 min at 4 °C to remove the debris. Assessing apoptosis-related proteins expression in the hippocampus could refer to PC12 cells.

We determined the levels of serotonin (5-HT), dopamine (DA) and norepinephrine (NA) in the hypothalamus using ELISA kits according to the manufacturer’s instructions. Absorbance was measured at 450 nm within 15 min of the reaction using a microplate reader.

### Statistical analysis

Data are expressed as the mean ± standard deviation. All statistical analyses were performed using one-way ANOVA followed by an LSD test for multiple comparisons using the SPSS 19.0 software (SPSS, Inc., Chicago). A *p*-value < 0.05 was considered significant.

## Results

### HPLC analysis of the chemical profile of EXD

**Figure 1.**
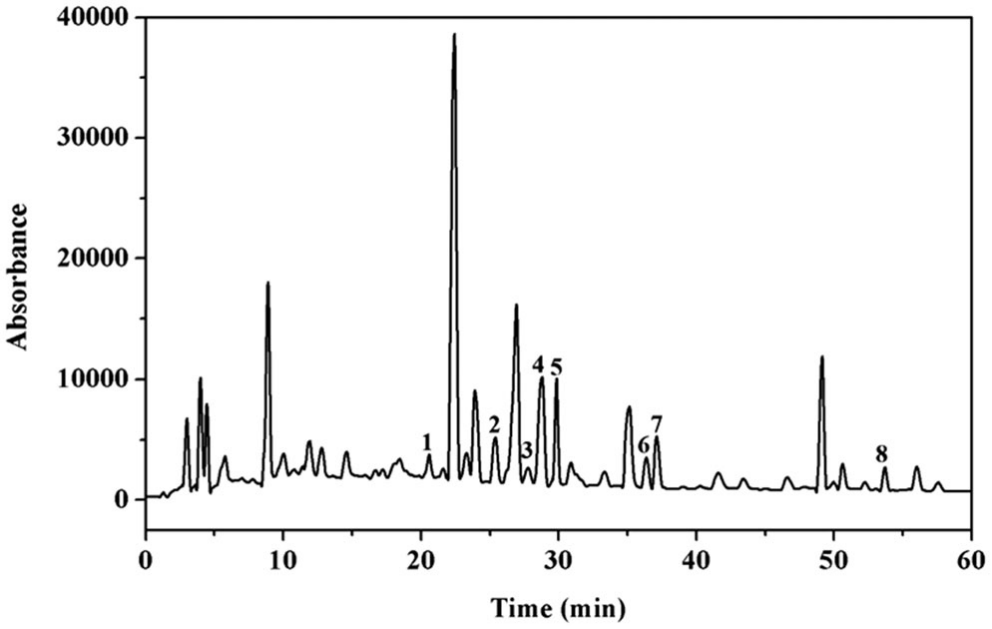
The chromatographic profile of Erxian Decoction (EXD); orcinol (**1**), mangiferin (**2**), 2, 6-dimethoxybenzoic acid (**3**), ferulic acid (**4**), curculigoside (**5**), berberine (**6**), epimedin C (**7**), and rubiadin (**8**). Peaks (**1**–**8**) were assigned based on the UV absorption and retention times of the authentic samples.

The composition of EXD was assessed using HPLC to provide chemical information regarding this formula. Qualitative analysis was conducted on the eight compounds [orcinol (**1**), mangiferin (**2**), 2, 6-dimethoxybenzoic acid (**3**), ferulic acid (**4**), curculigoside (**5**), berberine (**6**), epimedin C (**7**) and rubiadin (**8**)] in EXD using the optimized HPLC-photodiode array method. Each component of EXD was identified by comparing the retention time and UV spectra with the respective reference standards. The retention times of the eight marker compounds in EXD are shown in Figure 1.

### EXD inhibited corticosterone-induced cytotoxicity in PC12 cells

**Figure 2.**
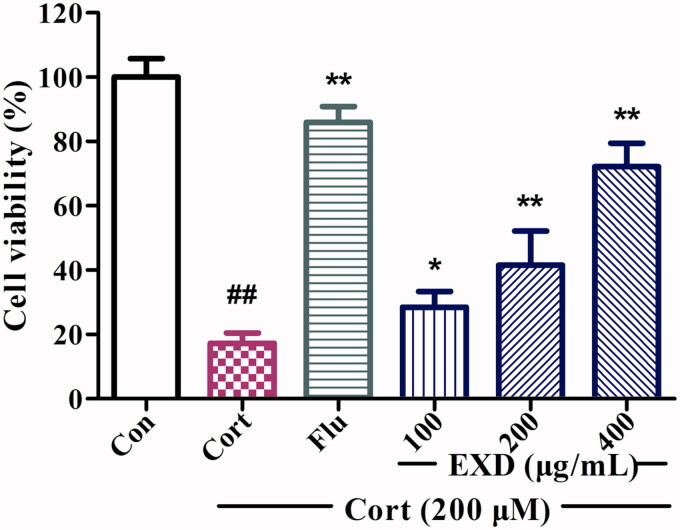
The effects of EXD on the viability of PC12 cells using MTT assay. Data are presented as the mean ± SD, *n* = 3. *p < 0.05 and **p < 0.01 versus Cort treatment; ^##^*p* < 0.01 versus control.

The protective effect of EXD against corticosterone-induced injury was assessed using the MTT assay. As shown in Figure 2, corticosterone (200 μM) severely caused cytotoxicity in PC12 cells, as indicated by the considerable decrease in cell viability. Treatment with EXD (100, 200 and 400 μg/mL) significantly increased cell viability, which suggested that EXD could effectively protect PC12 cells from corticosterone injury.

### Effects of EXD on the changes in cell morphology

**Figure 3.**
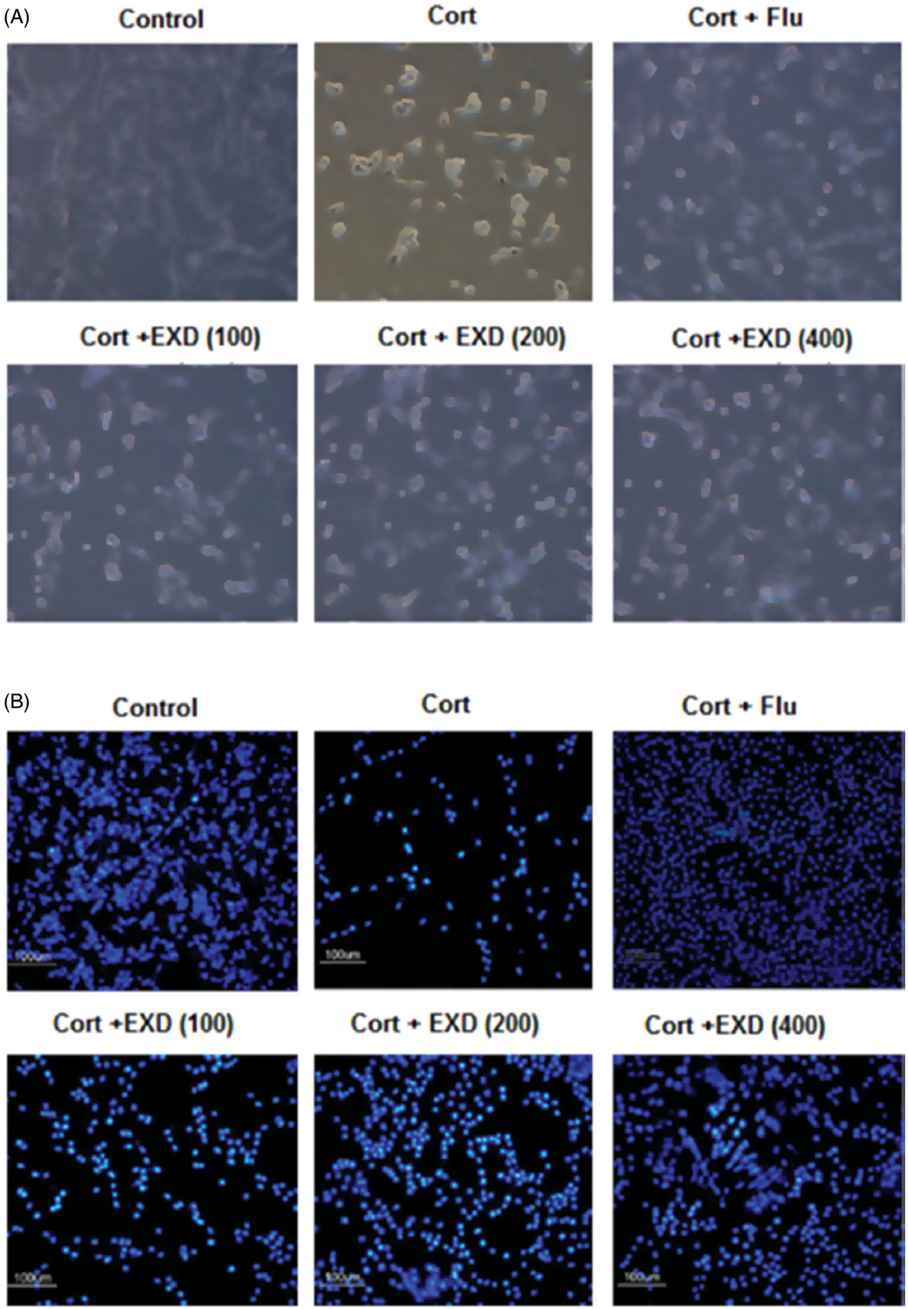
The effect of EXD on morphological changes in PC12 cells. A: Cells observed under an inverted microscope (200×). B: Hoechst 33258 staining for PC12 cells observed using fluorescence microscopy (200×). PC12 cells were pre-treated with fluoxetine or EXD for 1 h, followed by treatment with 200 μM corticosterone for 24 h.

In Figure 3(A), phase-contrast micrographs revealed that corticosterone (200 μM) caused a significant decrease in PC12 cell numbers compared to that in the control. Furthermore, typical apoptotic morphological changes, including cell shrinkage, pyknosis, apoptotic vacuoles, membrane blebbing and the formation of floating cells, were observed. Moreover, in the Hoechst 33258 staining assay, characteristic changes in apoptotic cell nuclei, such as chromatin condensation and fragmentation, were observed in corticosterone (200 μM)-treated PC12 cells under a fluorescence microscope (Figure 3(B)). All these morphological changes suggested that apoptosis occurred in corticosterone-treated PC12 cells. However, treatment with EXD (100, 200 and 400 μg/mL) effectively reversed these morphological changes.

### EXD inhibited LDH leakage in corticosterone-injured PC12 cells

**Figure 4.**
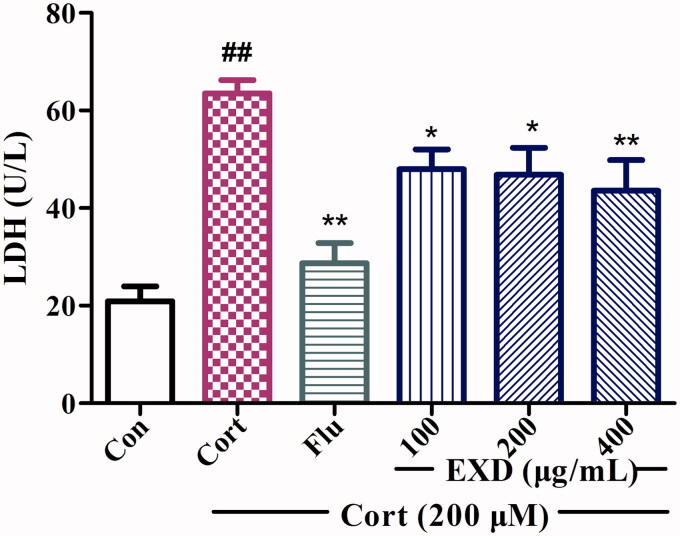
The effects of EXD on LDH release in PC12 cells. Data are presented as the mean ± SD, *n* = 3. *p < 0.05 and **p < 0.01 versus Cort treatment; ^##^*p* < 0.01 versus control.

LDH leakage is a marker of cell damage. The protective effect of EXD could also be assessed by analysing LDH leakage in cells. Figure 4 shows that corticosterone (200 μM) treatment led to a remarkable increase in LDH leakage in PC12 cells compared with that in the control group. Treatment with EXD (100, 200 and 400 μg/mL) significantly inhibited LDH leakage in cells. These results suggested that EXD could protect PC12 cells against corticosterone-induced neurotoxicity by inhibiting LDH leakage.

### EXD inhibited corticosterone-induced apoptosis in PC12 cells

**Figure 5.**
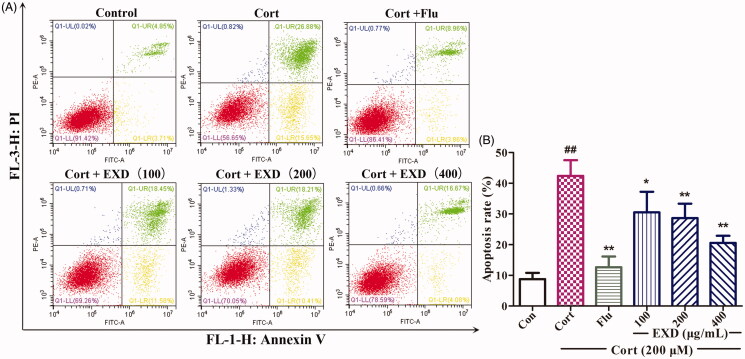
Flow cytometry analysis of apoptosis in PC12 cells measured with Annexin V-FITC and PI double-staining method. A: Representative dot plots of Annexin VFITC/PI staining; B: Bar graph indicating the percentage of apoptotic PC12 cells. Data are presented as the mean ± SD, *n* = 3. *p < 0.05 and **p < 0.01 versus Cort treatment; ^##^*p* < 0.01 versus control.

The anti-apoptotic effect of EXD on corticosterone-treated cells was determined by dual staining with Annexin V-FITC/PI using a flow cytometer. As shown in Figure 5, the percentage of apoptotic cells was 8.56% in the control group. After 24 h of exposure to corticosterone (200 μM), apoptotic cells increased significantly to 43.54%. On the contrary, the apoptosis rates of cells pre-treated with EXD (100, 200 and 400 μg/mL) were 30.02, 28.62 and 20.75%. These results demonstrated that EXD inhibited corticosterone-induced apoptosis in PC12 cells in a dose-dependent manner.

### EXD modified apoptosis-related proteins expression in corticosterone-treated PC12 cells

**Figure 6.**
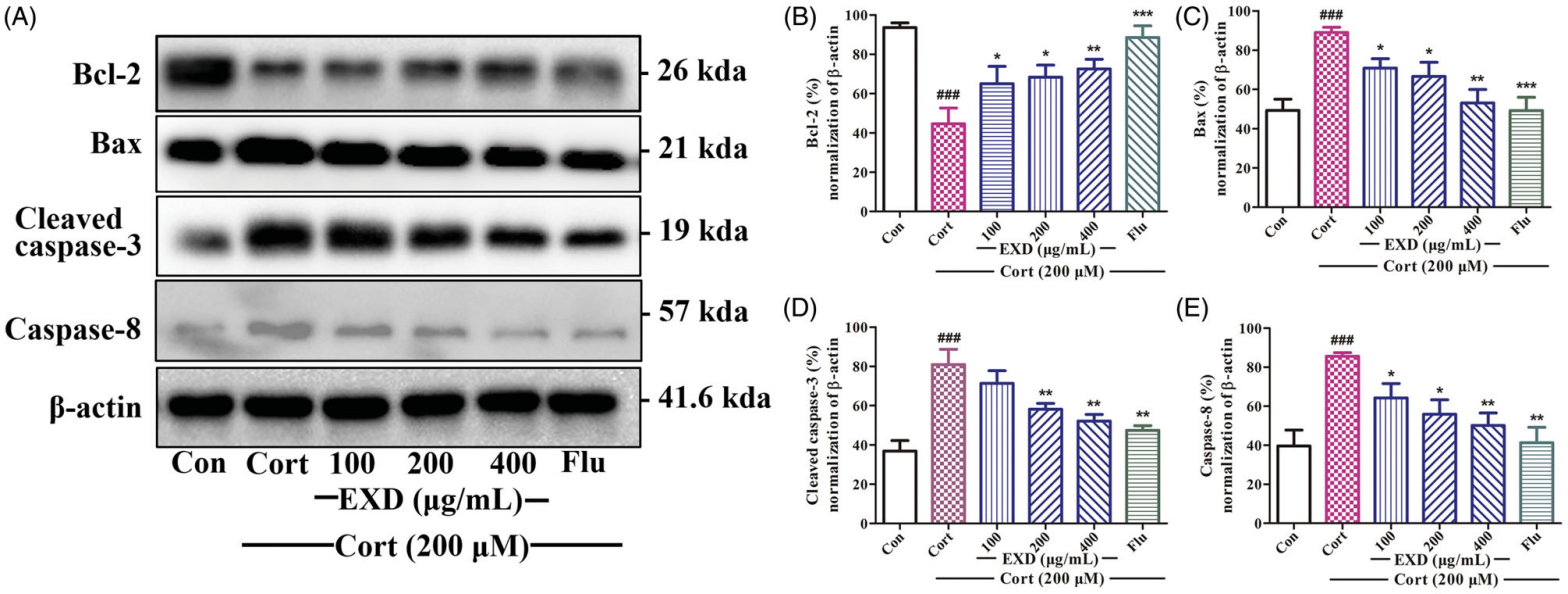
The expression of apoptosis-related proteins in corticosterone-treated PC12 cells. A: Western blotting analysis of Bcl-2, Bax, cleaved caspase-3, and caspase-8 using β-actin as a control; B: Densitometric quantification of the ratio of Bcl-2 to β-actin; C: Densitometric quantification of the ratio of Bax to β-actin; D: Densitometric quantification of the ratio of cleaved caspase-3 to β-actin; E: Densitometric quantification of the ratio of caspase-8 to β-actin; Data are presented as the mean ± SD, *n* = 3. *p < 0.05, **p < 0.01, and ***p < 0.001 versus Cort treatment; ^###^*p* < 0.001 versus control.

As shown in Figure 6(A–C), corticosterone (200 μM) significantly elevated the expression of the pro-apoptotic protein of Bax, and decreased the expression of the anti-apoptotic protein of Bcl-2 in PC12 cells, whereas EXD treatment (100, 200 and 400 μg/mL) significantly reversed these effects. Moreover, the expression of caspase-8 and cleaved caspase-3 were greatly up-regulated in corticosterone-treated PC12 compared with that in the control group (Figure 6(A,D,E)), whereas EXD treatment (100, 200 and 400 μg/mL) significantly decreased these changes. These observations indicated that EXD inhibited corticosterone-induced apoptosis in PC12 cells via a caspase-dependent pathway by modifying apoptosis-related proteins expression.

### EXD reduced the immobility time of mice in the despair models of the FST and TST

**Figure 7.**
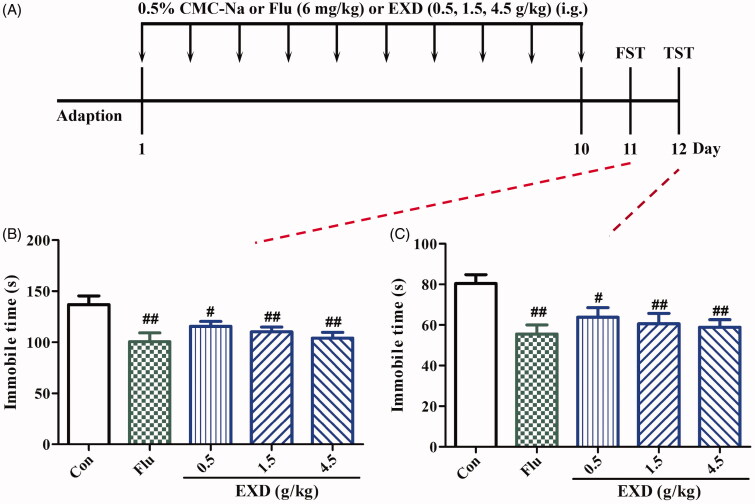
Effects of EXD on the immobility time in the despair model. A: Experimental schedule; B: FST; C: TST. Data are presented as the mean ± SD (*n* = 8). ^#^*p* < 0.05 and ^##^*p* < 0.01 versus control group.

Mice treated with EXD (0.5, 1.5 and 4.5 g/kg) for 10 days greatly reduced the immobilization time in the FST when compared with that in the control group (Figure 7(B)). In the TST, the control group also had much longer immobility time in contrast to the significantly reduced immobility time by mice treated with EXD (0.5, 1.5 and 4.5 g/kg) for 10 days (Figure 7(C)). Fluoxetine (6.0 mg/kg) significantly shortened the immobility time in both the FST and TST compared with those in the control group.

### EXD reversed reserpine-induced hypothermia, ptosis and akinesia in mice

**Figure 8.**
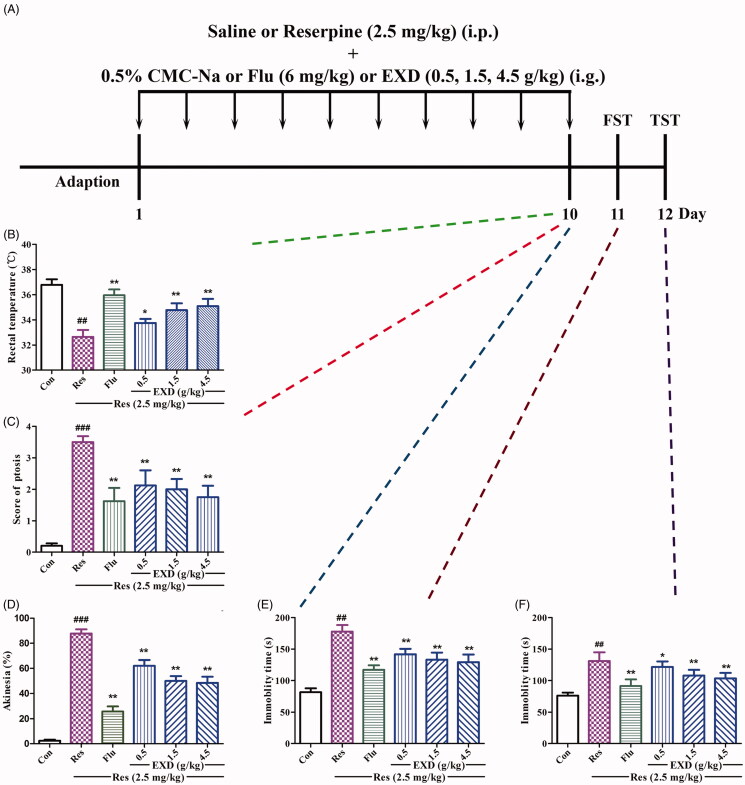
Antidepressant-like effects of EXD on mice in the reserpine-induced depression model. A: Experimental schedule; B: Antagonistic effect of EXD on reserpine-induced hypothermia in mice; C: Antagonistic effect of EXD on reserpine-induced palpebral ptosis in mice; D: Antagonistic effect of EXD on reserpine-induced akinesia in mice; E: Effect of EXD on immobility time in the FST of reserpine-treated mice; F: Effect of EXD on immobility time in the TST of reserpine-treated mice. Data are presented as the mean ± SD (*n* = 8). *p < 0.05 and **p < 0.01 versus Res treatment; ^##^*p* < 0.01 versus control.

The effects of EXD on reserpine-induced hypothermia, ptosis and akinesia are shown in Figure 8(B–D)). Mice treated with 2.5 mg/kg of reserpine alone had significantly altered hypothermia, ptosis and akinesia compared with the control group. EXD (0.5, 1.5 and 4.5 g/kg) treatment significantly diminished reserpine-induced hypothermia compared with the reserpine group. Also, EXD treatments (0.5, 1.5 and 4.5 g/kg) significantly decreased the scores of reserpine-induced ptosis and akinesia. Fluoxetine at a dose of 6.0 mg/kg significantly antagonised hypothermia, ptosis and akinesia induced by reserpine.

### EXD reduced immobility time of reserpine-treated mice in the FST and TST

As shown in Figure 8(E, F), a 10-day treatment of mice with reserpine (2.5 mg/kg) significantly increased the immobility time in both the FST and TST compared with those in the control group. EXD (0.5, 1.5 and 4.5 g/kg) treatment markedly reduced the immobility time in reserpine-treated mice in both the FST and TST compared with those in the reserpine group. Fluoxetine at a dose of 6.0 mg/kg significantly decreased the immobility time in the FST and TST experiments.

### The effect of EXD on the expression of apoptosis-related proteins in the hippocampus of mice, and improvement in neurotransmitter depletion induced by reserpine in the hypothalamus

**Figure 9.**
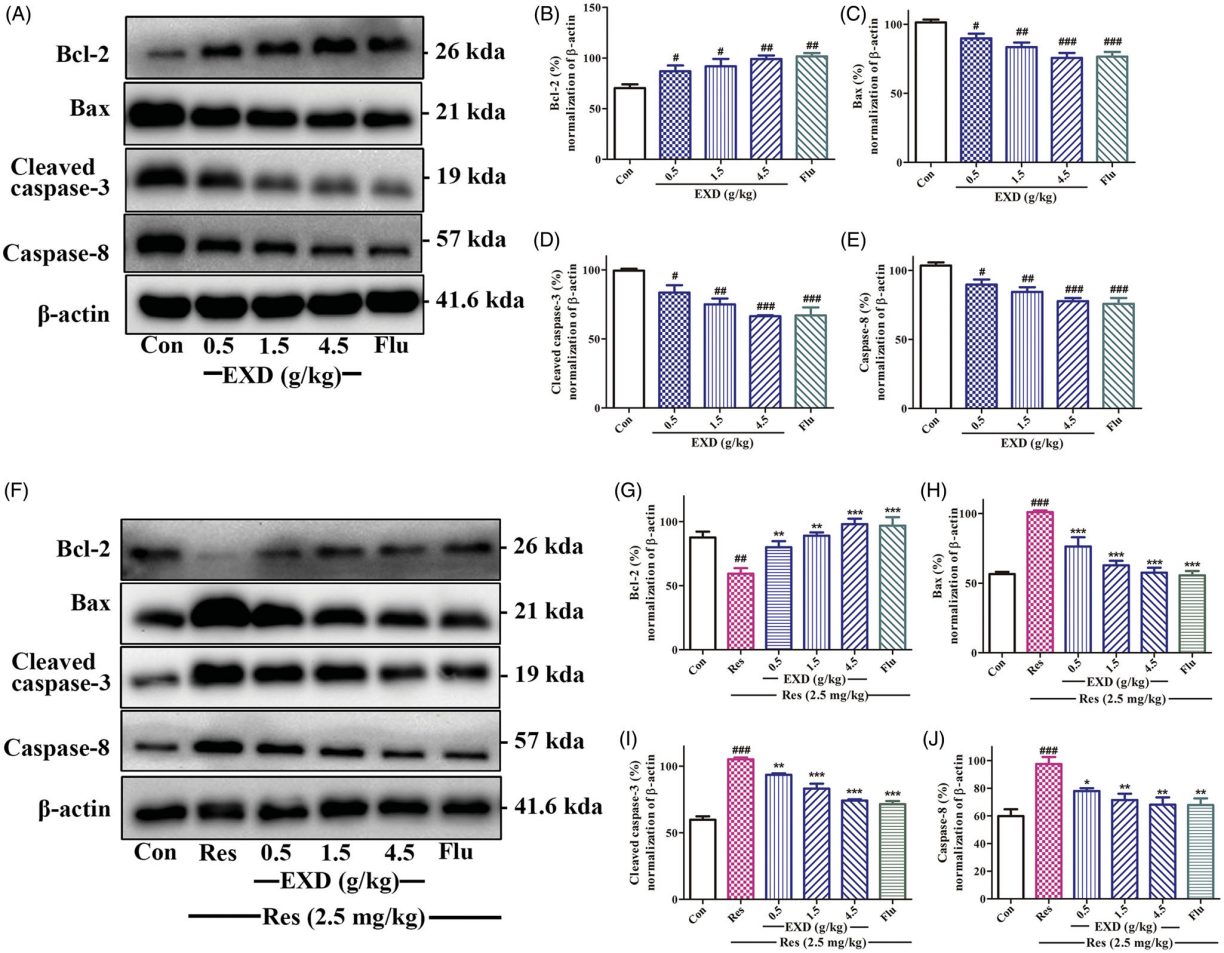
The expression of apoptotic proteins in the hippocampus in both the behavioural despair model and the reserpine-induced pharmacological model. A: Determining the levels of Bcl-2, Bax, cleaved caspase-3, caspase-8, and β-actin in the hippocampus of mice in the despair model with Western blotting. B–E: Ratios of Bcl-2, Bax, cleaved caspase-3, and caspase-8 to β-actin in the hippocampus of mice in the despair model. F: Determining the levels of Bcl-2, Bax, cleaved caspase-3, caspase-8, and β-actin in the hippocampus of mice in the reserpine-induced pharmacological model with Western blotting. G–J: Ratios of Bcl-2, Bax, cleaved caspase-3 and caspase-8 to β-actin in the hippocampus of mice in the reserpine-induced pharmacological model. Data are presented as the mean ± SD, *n* = 3. *p < 0.05, **p < 0.01, and ***p < 0.001 versus Cort treatment; ^##^*p* < 0.01 and ^###^*p* < 0.001 versus control.

In the behavioural despair model, western blotting showed that EXD treatment (0.5, 1.5 and 4.5 g/kg) significantly up-regulated the expression of the anti-apoptotic protein of Bcl-2 and down-regulated the expression of the pro-apoptotic proteins of Bax, caspase-8 and cleaved caspase-3 in the hippocampus of mice compared with those in the control group (Figure 9(A–E)). Findings with the highest dose of EXD (4.5 g/kg) were close to those found using fluoxetine.

Regarding the reserpine-induced pharmacological model, western blotting revealed that reserpine treatment significantly increased the expression of the pro-apoptotic protein of Bax and down-regulated the expression of the anti-apoptotic protein of Bcl-2 in the hippocampus of mice compared with the control group, while EXD treatment (0.5, 1.5 and 4.5 g/kg) reversed these effects (Figure 9(G,H)). In addition, reserpine treatment significantly up-regulated the proteins expression of caspase-8 and cleaved caspase-3 compared with the control group, whereas EXD treatment (0.5, 1.5 and 4.5 g/kg) reduced these changes (Figure 9(F,I,J)). These results suggested that the administration of EXD improved the development of depressive symptoms by protecting the hippocampus from reserpine treatment.

**Figure 10.**
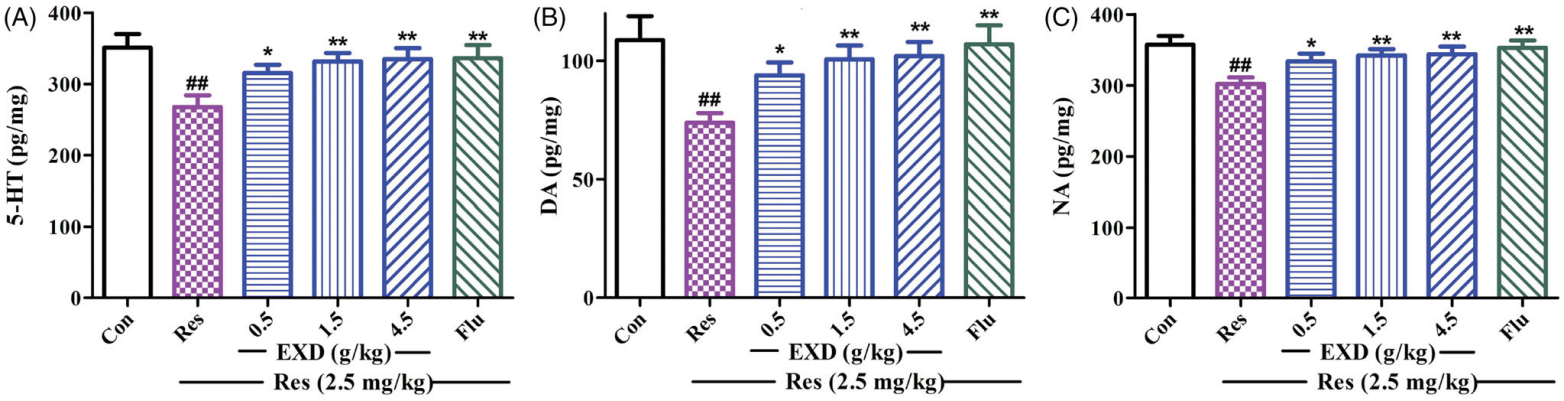
Effects of EXD on neurotransmitter levels in the hypothalamus of mice in the reserpine-induced pharmacological model. A: 5-HT levels in the hypothalamus; B: DA levels in the hypothalamus; C: NA levels in the hypothalamus. Data are presented as the mean ± SD (*n* = 8). *p < 0.05 and **p < 0.01 versus Res treatment; ^##^*p* < 0.01 versus control.

Several studies have shown that 5-HT, DA and NE are involved in the pathophysiological process of depression (Torres et al. 2003). To better evaluate the antidepressant effects of EXD, ELISA has been used to determine monoamine neurotransmitter levels. As shown in Figure 10, the levels of 5-HT, DA and NE in the hypothalamus of mice treated with reserpine were significantly reduced compared with the control group, whereas EXD treatment (0.5, 1.5 and 4.5 g/kg) reversed these changes. These results suggested that EXD treatment could inhibit the reserpine-induced depletion of monoamine neurotransmitters in the hypothalamus.

## Discussion

This study investigated the neuroprotective effects of EXD and its antidepressant potential. Our results revealed that EXD significantly increased the viability of PC12 cells pre-treated with corticosterone *in vitro*, markedly inhibited the corticosterone-induced apoptosis of PC12 cells in a dose-dependent manner by decreasing LDH leakage, and regulated the apoptosis-related proteins expression of Bcl-2, Bax, caspase-3 and caspase-8. *In vivo*, EXD effectively improved the depression-like behaviour of mice in despair and reserpine-induced models. The formula also inhibited cell apoptosis in the hippocampus, and reversed the depletion of monoamine neurotransmitters induced by reserpine.

Stress is the most important risk-triggering factor in the development of depression. Glucocorticoids (corticosterone in rodents, cortisol in humans) are pivotal neuroendocrine hormones in physiological response to stress (Gourley et al. 2013). Hypothalamic-pituitary-adrenal (HPA) axis dysfunction is a marker of neuroendocrine abnormality for depression (Thomson and Craighead 2008). Chronic stress leads to the hyperactivity of the HPA axis and overproduction of glucocorticoids (Herman et al. 2016). Sustained high levels of glucocorticoids activate glucocorticoid receptors and induce neuronal cell damage, as well as impair neurogenesis in the brain and cause depression symptoms (Zhu et al. 2006; Murray et al. 2008).

PC12 cells, a cell line with typical neuronal characteristics and high levels of glucocorticoid receptors, have been widely used as an *in vitro* model for both glucocorticoid-induced impairment of neuronal cells and their underlying molecular mechanisms (Terada et al. 2014). Increasing evidence has demonstrated that neurogenesis plays an important role in the onset and development of depression and in the therapeutic effect of antidepressants (Micheli et al. 2018). Bcl-2 and Bax are the anti-apoptotic and pro-apoptotic members of the Bcl-2 family, respectively. Bcl-2 prevents the apoptotic process through interaction with Bax to maintain mitochondrial membrane integrity, and blocks the release of cytochrome c from the mitochondria into the cytosol (Cory and Adams 2002). Caspase-8 is a key initiator caspase in the mitochondria that activates cell apoptosis, and caspase-3 acts as a critical effector in the apoptotic process (Sharifi et al. 2009). In this study, corticosterone (200 μM) greatly inhibited the proliferation of PC12 cells; EXD displayed a significant neuroprotective effect on corticosterone-injured PC12 cells, as indicated by the increased cell viability in the MTT assay. EXD also decreased cell LDH leakage and markedly inhibited corticosterone-induced apoptosis in PC12 cells in a dose-dependent manner by up-regulating the expression of anti-apoptotic protein Bcl-2 and down-regulating the expression of pro-apoptotic protein Bax. Additionally, EXD significantly inhibited the activation of caspase-3 and caspase-8 in corticosterone-treated PC12 cells. Therefore, EXD might exert its antidepressant effects via promoting neurogenesis.

In order to further assess the antidepressant effects of EXD *in vivo*, the behavioural despair model and reserpine-induced pharmacological model were used in this study. Despair is the core symptom of depression, and the FST and TST are the classic behavioural experiments widely used to evaluate this mood state (Bourin et al. 1983; Petit-Demouliere et al. 2005). The FST and TST-induced state of immobility in animals represents a condition similar to human depression; that is, animals under inescapable stress would become immobile and become sensitive to various antidepressant drugs (Yan et al. 2018). As expected, fluoxetine (6.0 mg/kg) decreased the immobility time in both the FST and TST in the present study, which suggests a potential predictive validity of the model. Oral administration of EXD (0.5, 1.5 and 4.5 g/kg) significantly reduced the immobility time in both the FST and TST compared to the control group, which indicates that EXD could alleviate a depressive mood. Our previous study revealed that orcinol glucoside of *C. orchioides* attenuated the immobility time of chronic unpredictable mild stress (CUMS)-induced rats in the FST and TST (Ge et al. 2014).

Disturbances in brain monoamine neurotransmitters play an important role in MDD. According to the monoamine theory by Schildkraut (1965), a functional decrease in the activities of brain amines, particularly in NA, DA and 5-HT, would result in depression. Reserpine depletes the neuronal storage granules of biogenic amines in the brains of rodents, and produces a clinically significant depression-like state (Minor and Hanff 2015). In response to reserpine, mice became hypothermic, with akinesia and drooping eyelids (Norn and Shore 1971). As a result, the reserpine-induced behaviours of hypothermia, ptosis and akinesia were observed in the present study, because of the depletion of monoamines stores. Antidepressants with a potential activation role in the central noradrenergic system inhibit these responses. In line with a previous report (Zhou et al. 2012), fluoxetine (6.0 mg/kg) significantly reversed reserpine-induced depression-like behaviour. In the present study, EXD (0.5, 1.5 and 4.5 g/kg) administration effectively mollified reserpine-induced hypothermia, ptosis and akinesia, and eliminated the reduction of monoamine neurotransmitters. These results suggest that the central monoamine neurotransmitter system in the brain might be implicated, at least partially, in the antidepressant-like effects of EXD.

Depression is caused by an injury to the brain or nerve tissues (especially hippocampal nerve tissues), which leads to the impairment of cognitive function, memory function and emotional regulatory functions (Jiang et al. 2019; Spanier et al. 2019; Wang et al. 2019). PC12 cells are derived from pheochromocytoma of the adrenal medulla of rats, and have the general characteristics of neuroendocrine cells. It is also a classic model of depression *in vitro* (Jiang et al. 2014; Tian et al. 2018). Therefore, the approach that protecting the nerve tissue is generally applied to the treatment of depression. In this study, EXD exerted neuroprotective effects *in vitro* and *in vivo* by regulating similar expression of apoptosis proteins; other tests also showed similar results.

TCM formula is an essential part of Chinese herbal medicines. It has been well accepted that the therapeutic effects of TCM formula are due to the interaction between individual chemical components in the herbs with multi-targets. In this study, the chemical profile of EXD was characterized using HPLC analysis. Eight compounds were assigned, with most of them being the main active components in the six herbal medicines (*C. orchioides*, *E. brevicornu*, *A. senensis*, *P. chinense*, *A. asphodeloides* and *M. officinalis*) (Figure 1). According to previous studies, curculigoside and orcinol glucoside are the representative compounds in *C. orchioides*. One recent study demonstrated that curculigoside prevents depression-like behaviours in a mouse learned helplessness model through increasing hippocampal Brain-derived neurotrophic factor (BDNF) (Yang et al. 2019). Our group previously reported that orcinol glucoside improved the depressive behaviour of CUMS rats by decreasing serum corticosterone levels and HPA axis hyperactivity, as well as increasing BDNF expression and ERK1/2 phosphorylation in the hippocampus (Ge et al. 2014). The flavonoids in *E. brevicornu*, such as icariin, displayed an antidepressant-like effects via its regulation of the central corticotropin-releasing factor system in chronic mild stress-induced rats (Pan et al. 2007, 2010). Ferulic acid, the main active compound in *A. senensis*, displayed an antidepressant-like effects in corticosterone-induced mice model (Zeni et al. 2017), and promoted neural progenitor cell proliferation in the hippocampus of corticosterone-treated mice *in vivo* (Yabe et al. 2010). Berberine, an alkaloid in the bark of *P. chinense*, improved the corticosterone-caused depression-like behaviour in mice by up-regulating BDNF expression in the hippocampus (Shen et al. 2016). Mangiferin, an anti-inflammatory C-glucosyl-xanthone present in the rhizome *A. asphodeloides*, exerted an antidepressant effect in lipopolysaccharide-induced mice model (Jangra et al. 2014). These active constituents of the individual herbs in EXD might account for the antidepressant-like effects of EXD to some degree. However, more work needs to be done in future.

## Conclusions

Our investigation revealed that the Chinese herbal medicine formula EXD had neuroprotective effects on corticosterone-injured PC12 cells *in vitro*. EXD significantly increased cell viability in the MTT assay, inhibited corticosterone-induced cell apoptosis in a dose-dependent manner by decreasing LDH leakage, and regulated the apoptosis-related proteins expression of Bcl-2, Bax, caspase-3, and caspase-8. *In vivo*, EXD appeared to have an antidepressant-like effects, as indicated by the reduced immobility time of mice in the FST and TST. EXD effectively antagonised reserpine-induced hypothermia, ptosis and akinesia in mice, and its antidepressant-like effects might be, at least partially, implicated in the regulation of the monoaminergic neurotransmitter system in the brain. These findings not only provide new insights into the neuroprotective potential of EXD, but also confirm its therapeutic benefits as an antidepressant agent in folk clinical application. Further studies are required to focus on the detailed antidepressant mechanism of EXD, and to identify the main active components in the formula responsible for the action.
